# Reflections on an Evidence Review Process to Inform the Co‐Design of a Toolkit for Supporting End‐of‐Life Care Planning With People With Intellectual Disabilities

**DOI:** 10.1111/hex.70062

**Published:** 2024-10-15

**Authors:** Elizabeth Tilley, Lorna Rouse, Irene Tuffrey‐Wijne, Rebecca Anderson‐Kittow

**Affiliations:** ^1^ School of Health, Wellbeing and Social Care, The Faculty of Wellbeing, Education and Language Studies The Open University Milton Keynes UK; ^2^ Centre for Applied Health and Social Care Research, Faculty of Health, Science, Social Care and Education Kingston University London London UK

**Keywords:** AGREE II, co‐design, evidence review, inclusive research, intellectual disabilities

## Abstract

**Introduction:**

There is growing recognition that healthcare inequalities faced by people with intellectual disabilities extend to their experiences at the end of life, resulting in calls for more inclusive research to help address these inequities. Our study aimed to address this through the co‐design of a toolkit for supporting end‐of‐life care planning with people with intellectual disabilities. To inform the co‐design process, we undertook an evidence review to identify existing tools, resources and approaches that were already being used in practice.

**Methods:**

Our evidence review comprised three components: (i) a rapid scoping review of the academic literature, (ii) a desk‐based search of the grey literature and (iii) an online survey to capture unpublished resources that were distributed to services, professionals, third‐sector organisations and family members. A longlist of existing materials was appraised using an adapted version of the AGREE II instrument, resulting in a shortlist that was shared with the co‐design team.

**Results:**

The evidence review played a critical role in the co‐design of a new toolkit of end‐of‐life care resources for people with intellectual disabilities. However, AGREE II proved to be limited for our purposes.

**Conclusions:**

The survey was particularly useful in helping us identify resources, tools and approaches in current use. We identified evidence review processes that served to support co‐design team activities and elements that were more problematic. We argue that evidence review practices might be enhanced to better aid co‐design activities in health and care research, particularly for studies involving people with intellectual disabilities.

**Patient or Public Contribution:**

This article reflects on an evidence review that was conducted as part of The Victoria and Stuart Project. People with intellectual disabilities were deeply involved at every stage of project design, delivery and dissemination. The project employed people with intellectual disabilities as members of the core research team. People with intellectual disabilities and family carers were members of the project co‐design team and the project Advisory Group. The evidence review process itself was led by academic members of the research team with contributions from colleagues with intellectual disabilities via the Advisory Group and core research team. The findings from the evidence review were used by the co‐design team to inform the development of an end‐of‐life care planning toolkit for people with intellectual disabilities.

## 1 Introduction

Inclusive research with people with intellectual disabilities has gained significant momentum in the 21st century, underpinned by a political stance of ‘nothing about us without us’. Co‐design has become an increasingly visible approach in intellectual disability health and care research in the UK context over the past decade and is a pre‐requisite for some funders [1]. This mirrors wider trends that advocate for greater patient and public involvement in the design, delivery and implementation of health and care research, including gathering and reviewing documentary evidence and the co‐development of new interventions, tools and guidelines [2, 3, 4]. Despite claims that we are now moving into the ‘second generation’ of inclusive intellectual disability research that prioritises outcomes over process, the time, complexity and care required to co‐research alongside colleagues with intellectual disabilities requires sustained reflection on methodological and theoretical issues [5]. To date, there has been limited reflection on how co‐designed projects tackle the process of evidence review and appraisal, and how they utilise its outcomes.

Collaborative research with people with intellectual disabilities has also engaged with topics that may be viewed as sensitive or even taboo (e.g., sexual and reproductive health, parenting and institutional histories), necessitating careful consideration of legal, ethical and emotional concerns [6, 7]. However, inclusive research focused on end‐of‐life care remains rare; one review found that study participants in this area were almost exclusively health and social care professionals [8]. Another review found that although the importance of including people with intellectual disabilities in end‐of‐life decisions was emphasised, only two out of 10 studies described decision‐making processes that involved people themselves [9]. Co‐researching this difficult topic area with people with intellectual disabilities is an important step towards addressing inequities in end‐of‐life care provision for people with intellectual disabilities, such as lack of care coordination and reduced access to specialist palliative care services and opioid analgesia [10]. But there is little knowledge or guidance on how to do this well.

The Victoria and Stuart Project (NIHR202963) aimed to improve personalised end‐of‐life care provision for people with intellectual disabilities by co‐producing a toolkit of end‐of‐life care planning (EOLCP) approaches and resources that are beneficial to people with intellectual disabilities and workable within adult health and social care services. A co‐design group including members with and without intellectual disabilities developed a toolkit comprising three new resources and links to existing resources. These were informed by an evidence review and empirical data collected in the first stage of the project [11]. This approach aligns with evidence suggesting that co‐design may positively impact researchers, practitioners, research processes and outcomes, such as usability and applicability of research, skills development for co‐researchers, improving the efficacy of interventions and strengthening pathways to impact [12, 13].

The first aim of the evidence review was to create an inventory of currently available tools, resources and approaches to support EOLCP with people with intellectual disabilities published in the international academic literature and used by voluntary and social care sector organisations in the United Kingdom. The second aim of our evidence review was to produce a shortlist of the most promising outputs for consideration by a co‐design group that included nine people with intellectual disabilities, as well as family carers and professionals. More specifically, the co‐design group wanted to know: (i) what tools, resources and approaches (with associated guidance) had already been produced; (ii) how the material had been produced (context and stakeholders); (iii) whether the material was being used in practice; and (iv) what appeared to be working well and what were the limitations. The co‐design team needed to identify and address current gaps, while learning as much as possible from previous attempts to design and implement resources on EOLCP for people with intellectual disabilities. Critically, the co‐design team was determined not to ‘re‐invent the wheel’ by inadvertently duplicating previous efforts.

Evidence reviews are identified as a key step in Armstrong et al.'s framework for enhancing clinical practice guidelines through continuous patient engagement [14]; however, they are often an under‐reported component of inclusive research with people with intellectual disabilities. The purpose of this article is to reflect upon how an evidence review informed the co‐design process in the context of co‐researching alongside people with intellectual disabilities. Specifically, we consider the added value of evidence reviews for co‐design activities, considering which components of the review process were most useful, while acknowledging some notable limitations. This article does not report the outcomes of the evidence review but focuses instead on the practices involved in inventorying and appraising existing EOLPC resources and approaches for people with intellectual disabilities. We detail the process undertaken to scope out and assess the quality, suitability and relevance of tools, resources and approaches that could be utilised to inform the co‐design of an EOLCP toolkit. In doing so, we reflect on the specificities of evidence review practice in the context of inclusive co‐design activities. This includes a discussion on the operationalisation of a prioritisation and consensus exercise utilising the AGREE II (Appraisal of Guidelines, Research and Evaluation) instrument to appraise the materials identified through a rapid scoping review of the literature, a search of the grey literature and a survey. Although our evidence review process drew heavily on established academic approaches, it also required us to be flexible and open to a level of experimentation, for example responding to the recommendations of our Advisory Group regarding engagement with stakeholders and revising our approach to quality appraisal to support the wider project aims and the needs of the co‐design group.

## 2 Evidence Review Methodology

Our evidence review was designed to address the following research question:

*What tools, resources and approaches have been developed, adapted or used to support end‐of‐life care planning with people with intellectual disabilities in the United Kingdom and internationally?*

**Figure 1:**
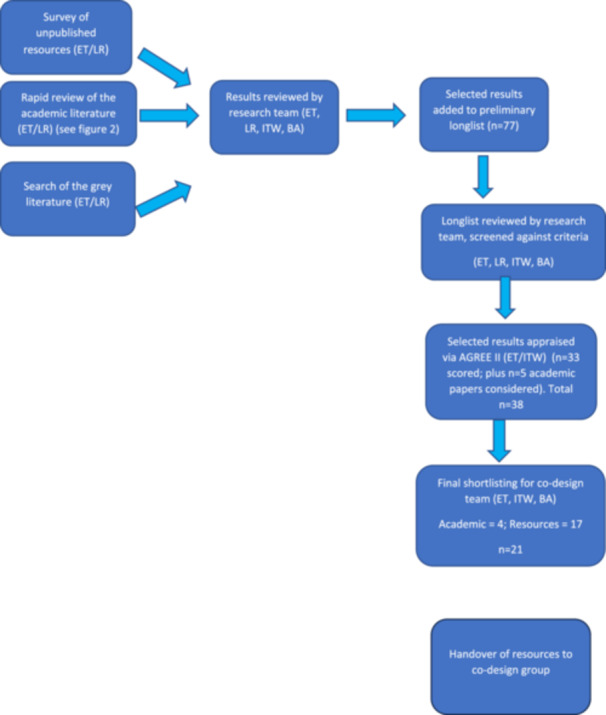
Evidence review steps.

Our evidence review methodology comprised three components, which are detailed later: (i) a rapid scoping review of the international academic peer‐reviewed literature, (ii) a rapid scoping review of the grey literature and (iii) a survey to elicit unpublished resources used by stakeholders in the UK intellectual disability social care and voluntary sectors. Resources gathered were reviewed by the research team with the aim of agreeing on a longlist of resources that could be assessed for quality and suitability using an adapted AGREE II instrument [15, 16]. Figure 1 outlines the steps in the evidence review.

Throughout all stages of the process, the authors of this article elicited feedback and sought advice and guidance from the wider research team and Advisory Group. Where input from colleagues and advisors with intellectual disabilities was needed (particularly for resources developed specifically to be accessible), the process was adapted to enable their input at the co‐design stage. They were presented with extracted aspects of the materials/proformas that covered identical topic areas (e.g., ‘Where do you want to die?’) and were then invited to use or complete the forms so that researchers could observe how they engaged with the materials. Their specific feedback on what they liked or disliked informed whether other similar materials, or parts thereof, were discarded or further tested.

### 2.1 Rapid Scoping Review of the Academic Literature

A rapid scoping review of the academic literature was adopted to meet the evidence review's aims to be inclusive and exploratory in nature, to capture a broad range of evidence drawn from diverse sources and to be undertaken in a timely fashion to inform the co‐design team's activities [17, 18]. The design was informed by the Joanna Briggs Institute (JBI) methodological guidance for scoping reviews [18] and by Arksey and O'Malley's [19] five‐step framework for scoping reviews.

The review protocol was developed using the Population, Concepts and Contexts framework (PCC) [18]. Search terms (Table 1) were selected through familiarisation with the literature and collaboration with the wider research team, which included experts in the fields of end‐of‐life care and healthcare for people with intellectual disabilities. Search terms were chosen to be inclusive of varying terminology internationally, over time and within different fields. The search strategy was refined through pilot searches of the literature. Databases were chosen to reflect the breadth of disciplines that may cover research on EOLCP for people with intellectual disabilities, including medical, psychology, public health and health and social care, and were refined based on pilot searches.

**Table 1:** Search terms.

Intellectual disability terms	(‘developmental* disab*’) OR (‘developmental delay’) OR (‘developmental disorder’) OR (‘learning disab*’) OR (‘intellect* disab*’) OR (‘intellect* disorder*’) OR (‘intellect* impair*’) OR (‘intellect* handicap*’) OR (‘learning difficult*’) OR (‘learning difficult*’) OR (‘cognitive disab*’) OR (‘mental* retard*’) OR (‘mental* handicap*’) OR (‘intellectual* handicap*’)
End‐of‐life terms	(‘end of life’) OR (end‐of‐life) OR (palliative) OR (death) OR (dying) OR (terminal) OR (bereavement) OR (life‐limiting) OR (‘advance care’)

Searches were made for international literature published in English from 2007 to 2022. The start date of 2007 was chosen to encompass literature published following the publication of Mencap's *Death by Indifference* report [20], which prompted a growing interest in the experiences of people with intellectual disabilities at the end of life in the United Kingdom, including annual national reviews of the deaths of people with intellectual disabilities [21, 22, 23].

**Figure 2:**
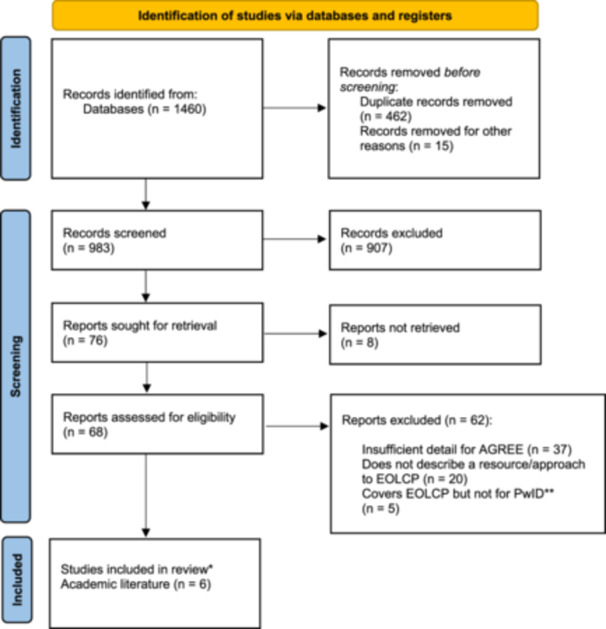
PRISMA 2020 flow diagram for new systematic reviews that included searches of databases and registers only. *Papers via electronic database searches that were included in the preliminary longlist of resources. **People with intellectual disabilities from: Page et al. [25]. For more information, visit: http://www.prisma-statement.org/.

The study selection process is reported in accordance with the Preferred Reporting Items for Systematic Reviews and Meta‐Analyses Extension for Scoping Reviews (PRISMA‐ScR) Checklist (see Figure 2 for an overview of the screening process adopted) [24]. Details of the inclusion/exclusion criteria used are provided in Table 2. Reasons for exclusion were as follows: insufficient detail for quality assessment via AGREE II (*n* = 37); does not describe a resource/approach to EOLCP (e.g., describes end‐of‐life care delivery rather than planning; *n* = 20); does not include people with intellectual disabilities (e.g., focuses on older people or people with dementia; *n* = 5).

**Table 2:** Inclusion and exclusion criteria.

Inclusion criteria	Exclusion criteria
Published in English. Published/made available from January 2007. Reports a literature review concerning resources^a^ or approaches^b^ to EOLCP and EOL decision‐making for PwLD. Reports empirical research concerning resources or approaches to EOLCP and EOL decision‐making for PwLD. Reports a case study concerning resources or approaches to EOLCP and EOL decision‐making for PwLD. Policy or practice guidance which reports resources or approaches to EOLCP and EOL decision‐making for PwLD. Reports resources or approaches to EOLCP and EOL decision‐making for PwLD through other types of publication, including editorials, websites, opinion pieces and study protocols. Concerns resources or approaches to EOLCP and EOL decision‐making that have been designed or adapted for adults (18+) with learning disabilities^c^ (including mild, moderate, severe/profound LD, severe communication difficulties and lack of capacity).	Publication is not available in English. Published/made available before January 2007. Concerns resources or approaches to EOLCP and EOL decision‐making that have not already been adapted and/or used for PwLD (including resources designed/adapted for people with autism who do not have a learning disability). Concerns resources or approaches to EOLCP and EOL decision‐making designed for children with learning disabilities (under 18). Concerns EOLC delivery, experience or staff training with no consideration of planning. Does not meet any of the inclusion criteria.

^a^ Resources include EOLC plans, EOL decision‐making tools and materials.

^b^ Approaches include policies and models of EOLCP.

^c^ Following the DoH definition of learning (intellectual) disability, which includes a reduced ability to understand new or complex information, to learn new skills and to cope independently (impaired social functioning) that starts before adulthood and has a lasting effect on development, this definition encompasses people with a broad range of disabilities and covers adults with autism who also have learning (intellectual) disabilities but not those who have a specific learning difficulty, such as dyslexia [26].

The rapid scoping review of the academic literature returned papers that described approaches to EOLCP, made recommendations for effective EOLCP and referred to approaches to EOLCP within descriptions of the experience of end‐of‐life care. The wider research team, comprising four academics (all with expertise in intellectual disability and two with specific knowledge of EOLCP), met to review the findings of the literature review to identify relevant papers to be included in the longlist of resources/approaches. Following group discussion, six papers were selected that described resources/approaches focused on staff training and communication in relation to end‐of‐life care, including planning. A manual search of the references of these papers returned one further paper that was screened and approved, resulting in a total of seven papers going forward to the preliminary longlist.

### 2.2 Review of the Grey Literature

A grey literature search was performed using the search terms mentioned in Table 1. Two searches were made using Google to split multiple synonyms for the term ‘intellectual disability’ into manageable searches. To complete the review within the available time period, a limit was set to the first five pages of search results from Google. This involved following up 50 links for each search (100 in total), which led to the screening of 28 potentially relevant resources. Further sources of grey literature included material known to members of the wider research team and the inclusive project advisory group. The grey literature search aimed to identify tools, resources or approaches to EOLCP for people with intellectual disabilities outside of the academic literature, and screened for relevance to the research question. Reasons for decisions to include/exclude were recorded. Lorna Rouse conducted the initial search and screening with input from Elizabeth Tilley. Twelve items were identified through the screening process. After reviewing a summary of the results of the grey literature search, we selected eight resources to be added to the preliminary longlist.

### 2.3 Survey to Identify Unpublished Resources

We complemented our reviews of the academic and grey literature by running an online survey targeted at those who support EOLCP for people with intellectual disabilities in the United Kingdom. The utility of scoping reviews to policymakers, practitioners and service users can be enhanced when combined with consultation exercises that engage stakeholders [19]. Online surveys have previously been found to be valuable for collecting data from those who work with people with intellectual disabilities on end‐of‐life‐related topics [27]. To capture resources and guidance currently in use that may as yet be unpublished, an online survey was conducted of social care providers, voluntary sector organisations working with people with intellectual disabilities and their families and other stakeholders who may be involved with EOLCP for people with intellectual disabilities in the United Kingdom.

A short online anonymous survey was devised by members of the research team (including research partners from health and social care practice) using Microsoft Forms to produce the survey and to collect responses. It was aimed at all those who are involved in, or concerned with, the care of people who are supported by intellectual disability services (in residential or supported living settings). This included direct support staff, service managers, health and social care professionals, family members and policymakers (see Appendix S1 for details of the survey questions and numerical responses). A participant information sheet and a link to the online survey were circulated by the project's partner organisations, the wider project team, the advisory group and their contacts through email, social media calls and newsletters targeting the voluntary sector, service providers, palliative care providers/educators, intellectual disability nurses, faith community leaders and family members of people with intellectual disabilities.

There were 95 responses to the survey in total, submitted by a range of health and social care professionals and some family members. Respondents described a mix of (i) EOLCP resources designed specifically for use with or by people with intellectual disabilities, (ii) generic EOLCP resources used to support people with intellectual disabilities and (iii) resources that were not specifically designed for EOLCP but that had been used to support EOLCP with people with intellectual disabilities, for example, resources to support communication, assess capacity or manage pain. Sixty‐two distinct resources were added to the preliminary longlist as a result of survey responses, survey follow‐up and contact from members of the research team. The survey proved to be a highly effective method in supporting our attempts to identify materials and resources that were being used in practice.

### 2.4 Appraisal of Resources: Using Agree II

The material selected for the longlist was assessed for quality using an adapted version of the appraisal tool AGREE II to assess methodological rigour, transparency, relevance and suitability of identified resources and approaches [15, 16, 28]. The AGREE II instrument provides a set of systematically developed statements for healthcare guidelines with 23 items across six quality domains:

1. scope and purpose,
2. stakeholder involvement,
3. rigour of development,
4. clarity of presentation,
5. applicability,
6. editorial independence.

AGREE II also includes a final ‘overall utility’ question at the end. In the absence of a specific tool to assess the quality of health or social care resources for people with intellectual disabilities, the AGREE II instrument appeared to provide a sufficiently broad and flexible framework to help us decide which tools, resources and approaches would best inform the co‐design stage of the wider study [28]. In addition, the instrument has previously been found useful to assess the quality of guidance on end‐of‐life care [29]. Our research presented a unique opportunity to apply the tool in the context of an evidence review process designed to inform the co‐design activities of a team of researchers with and without intellectual disabilities.

### 2.5 Running the Longlist Through Agree II

In preparation for the appraisal of resources, a preliminary longlist was produced combining the findings of the academic and grey literature reviews and the survey. This comprised a total of 77 resources, with details captured in a summary table, shared among the project team. The team met to read through each of the resources and make decisions on inclusion for quality appraisal based on consideration of relevance to the review's aims (i.e., does the item focus on planning end‐of‐life care rather than on end‐of‐life care delivery?) and duplication (the team aimed to exclude multiple variations on one style of document). This process refined the longlist of resources for appraisal via AGREE II to 33 resources/tools and 5 peer‐reviewed papers.

Reviewers prepared by following the AGREE II online training tool [30] and followed instructions for using AGREE from the user manual [28]. At this point, we discussed potential adaptions to AGREE II, notably the insertion of a small number of new and amended questions within specific domains. One member of the team (Lorna Rouse) piloted the instrument with these adapted questions using a selection of resources. We then met to discuss issues raised through the pilot activity and agree adaptations to AGREE II questions and the rating process to ensure the best fit for the purposes of our study. To extract data on the rating process, a spreadsheet was developed recording the relevant information/responses for each of the 33 items under headings for AGREE II's domains with subheadings for their corresponding questions. Efforts were made to gather any related information on resources which did not appear in the original document, for example, details on the development of resources or stakeholder involvement through online searches and emails to authors. The five academic articles were not scored using the adapted AGREE II framework, but the AGREE II framework was used to inform discussions about the potential utility of those academic articles for the co‐design group's work.

The appraisal exercise was led by one academic reviewer (Elizabeth Tilley), with final scores discussed and agreed upon by two other academic members of the team (Irene Tuffrey‐Wijne and Rebecca Anderson‐Kittow). A Word table was created to support the shortlisting process, which summarised the following information for review by the team: name and author, type of material, total score out of 161, percentage score for Domain 1 (scope and purpose) and percentage score for Domain 4 (clarity of presentation). These domains were deemed by the wider research group to be most relevant for the co‐design process, which is why we decided to specify the scores for these components.

## 3 Results

### 3.1 Shortlisting for the Co‐Design Group

AGREE II is most commonly utilised to assess the quality of clinical guidelines to make recommendations for their use. We drew upon and adapted AGREE II to assess the quality and suitability of material to inform research co‐production activities and support the work of our project co‐design group in the development of a new EOLCP tool. Thus, unlike many studies that report on their application of AGREE II for the purpose of making clinical recommendations [31], we are not presenting detailed scoring for individual resources in this article. Rather, we focus on how this process informed our decisions about which tools/resources/approaches to shortlist for our co‐design team.

A research team meeting was held to discuss the AGREE II results, which led to 15 of the 33 non‐academic items being included on the shortlist. The material was shortlisted where it scored at least 40% overall and 75% in either or both Domain 1 (scope and purpose) and Domain 4 (clarity of presentation). Two resources were shortlisted where it was agreed that their ‘overall utility’ superseded their low overall scores (under 40%) and their lower scores for Domains 1 and 4 (i.e., where these scored below 75%). One further resource that could not be satisfactorily run through the AGREE II process due to its format and lack of contextualising information (an online video) was added due to its overall utility and accessibility. A final resource that came to light very late in the process but was assessed as having significant relevance and utility was also included. The team included five peer‐reviewed articles in the shortlist due to their specificity and relevance based on the AGREE II domains and sub‐questions.

The ‘overall utility’ of some of the longlisted material was difficult to assess confidently without the input of co‐researchers with intellectual disabilities, which was planned for the next stage (co‐design) using shortlisted material only. This was particularly the case for easy‐read resources. While acknowledging that the evidence underpinning claims that easy‐read information leads to improved healthcare outcomes for people with intellectual disabilities is contested and limited [32], we were mindful that many resources returned through our search that had been designed for use with people with intellectual disabilities were in easy‐read formats. This finding, in conjunction with a preference expressed by our co‐design team to see some examples of easy‐read EOLCP material, informed our decision to shortlist four easy‐read resources, despite low scores.

At the end of our evidence review, we produced a shortlist of 21 items that comprised 17 resources and 4 academic articles. The resources were published between 2007 and 2022 and comprised a mix of guidance (for staff, providers or commissioners), staff training packs and resources/templates/tools (which may or may not have attached guidance) to be used by/with people with intellectual disabilities. The downloaded or hardcopy versions of resources and summaries of the academic articles were shared with the project's co‐design team, which conducted their own quality review to identify strengths, weaknesses and gaps in existing resources to support the development of a new EOLCP tool.

### 3.2 Views of the Co‐Design Group on the Short‐Listed Material

At the co‐design stage, the nine researchers and advisors with intellectual disabilities considered the materials aimed at supporting their involvement in EOLCP in two ways: (1) trying them out, with researchers with and without intellectual disabilities noting how they responded, and (2) giving considered feedback on the pros and cons of the material. Thisr was especially helpful for the easy‐read forms, which none of the co‐design members felt able to complete, with comments including ‘I would put that in the bin’ and ‘I probably wouldn't use that one’. This corresponds with wider critiques of easy‐read healthcare resources, which points to the limitations of adapted materials in aiding understanding, particularly when this has not been tailored towards people's individual information requirements and communicative support needs [32]. It meant that some shortlisted easy‐read materials were not in the end reviewed in depth by the group, as the initial negative verdict and clear justification for rejection were extended by our co‐researchers to all easy‐read forms. Conversely, EOLCP resources that did not use words (e.g., Talking Mats and Books Beyond Words) were welcomed by the group, who commented ‘I liked that there was no pen and paper’ and a consensus that ‘we want more pictures like these’.

Materials aimed at staff, including general guidance and training materials, were presented to the co‐design members without intellectual disabilities (family carers and professionals), in conjunction with the results of stakeholder focus group discussions around EOLCP that had been held alongside the evidence review. The group members also received links to the material by email, but no one reported looking through them in detail. It appeared too onerous a task, as many of these documents were lengthy and somewhat overwhelming. Rather, these resources were discussed in project meetings in more general terms. This process led to the identification of gaps in the provision of easy‐to‐digest and attractively presented staff training materials. The content needed for staff guidance and training was subsequently elicited from members of the co‐design group (with and without intellectual disabilities) through brainstorming and discussing staff skills, attributes, facilitators and barriers to EOLCP. The research team cross‐referenced this with the content and design of the shortlisted guidance and training resources before designing and testing new resources to fill the gaps.

### 3.3 Suitability and Usability of Agree II for Our Review

Critical to this evidence review process was our adaptation of the AGREE II instrument to include new and amended questions relevant to EOLCP with people with intellectual disabilities within the existing AGREE II domains. Key considerations in adapting AGREE II for our study included the need to capture the usability of the tools, resources and approaches for different groups of people with intellectual disabilities, including those with severe/profound intellectual disabilities, and their relevance to different circumstances and care settings. In contrast to other studies that have tended to foreground rigour of development [33], we agreed to prioritise tools, approaches and resources that scored particularly high in the domains of ‘scope and purpose’ and ‘clarity of presentation’ (Domains 1 and 4). This was a pragmatic decision informed by the priorities of the co‐design group, which recognised that many of the items on our longlist had not been developed by academics, nor produced as a result of empirical research projects. It was critical that our appraisal exercise did not result in the exclusion of resources that had the potential to be highly useful for the co‐design team because those resources had not described their underpinning methodology or development processes in significant detail. More specifically, we were mindful that without such weighting in favour of specific domains, we risked excluding a number of accessible resources, which were of particular interest to the co‐design group.

As discussed earlier, the AGREE II instrument includes the option of a final global question focused on the overall utility of the material that can be deployed to override an otherwise low score where appropriate. The team agreed to use this final question to help address potential dilemmas arising when a particular tool/resource/approach appeared particularly innovative or relevant to the co‐design group, but which had produced a low score. Although this feature of AGREE II has been criticised for its inherent subjectivity and lack of standardised application [33], it proved useful for the purposes of our evidence review, which was driven by the requirements of the co‐design team. It became evident during this study that the question on ‘overall utility’, which could override other scores, was crucial. Some of the materials that went on to be most highly regarded by people with intellectual disabilities had low AGREE II scores. However, the academic team acknowledged that although we remained in dialogue with the wider inclusive research team during the evidence review, judgements based on ‘overall utility’ were ultimately made by academic researchers and not people with intellectual disabilities. As such, this final global question reframed in more accessible language (‘Do you like it?’) was taken forward into the co‐design stage to prompt discussions about whether an item should remain in or out of scope for the toolkit.

## 4 Discussion

The Victoria and Stuart Project pushed boundaries regarding the inclusion of people with intellectual disabilities as university‐employed researchers and leading contributors to the co‐production of new resources [11]. This aligns with broader agendas regarding the democratisation of research, and ambitions to address the ableist logic that characterises many academic institutions [34, 35]. However, as a project team, we made a decision to structure the research to play to people's respective strengths and interests, mindful that people with intellectual disabilities are often resistant to traditional academic methods or roles [36], preferring instead to ‘steer things according to their motivations’ [37]. In practical terms, this meant prioritising the time of people with intellectual disabilities during the co‐design phase of the project, leaving academic researchers to lead the underpinning evidence review. At project completion, we have reflected on the impact of this decision, the efficacy of our evidence review methods and the review's added value (or otherwise) in terms of supporting the co‐design group's objectives.

Overall, we stand by our decision to appoint academic researchers to lead the evidence review process. Many of the tasks involved in evidence reviews are weighted towards a particular set of skills (e.g., scanning abstracts at speed, producing detailed spreadsheets and reviewing long publications for relevant information) and require a level of time and resources that would have been difficult to achieve with our co‐researchers in the context of our project schedule and budget. The difficulty of this task was not limited to co‐design members with intellectual disabilities; staff and family carers also felt unable to fully assess all shortlisted materials. However, on reflection, we do believe that we missed an opportunity to engage our co‐researchers in the development of a bespoke critical appraisal tool at an early stage of the project. Our methods enabled us to identify a wide range of existing resources, with the sector survey being a particularly useful strategy for our team (a method we would use again in future evidence reviews of this kind). However, the AGREE II instrument, although useful in lieu of anything more suitable, ultimately proved to be limited for our purposes. For example, it was not compatible with or relevant to all the resources and approaches identified. Although it was possible to make some adaptations within existing domains, AGREE II may not include all the points that people with intellectual disabilities consider important to assess the quality, suitability or relevance of a resource. We also found that it could not be sufficiently flexed to include some multimedia content or material that was highly visual, which was of particular interest to our colleagues with intellectual disabilities. Our experience of using AGREE II indicated that there is room for the development of an appraisal tool specifically designed for quality assessment of health and care resources for people with intellectual disabilities, which reflects the needs and concerns of people with intellectual disabilities and their carers and which has the flexibility to encompass a range of resources beyond clinical guidelines. In our view, the development of such a tool can only be achieved in collaboration with people with intellectual disabilities and other invested parties (e.g., family members and health and social care professionals). The timescales we were working on our research did not afford us the opportunity to develop a new tool in response to the challenges we experienced using AGREE II. For that reason, we persevered with AGREE II, finding ways to draw on its key tenets to produce a shortlist that would meet the needs of the co‐design group.

Reflecting at the end of the project and following the launch of a new set of co‐produced resources to support EOLCP for people with intellectual disabilities [11], we are persuaded that the evidence review made a significant contribution to the co‐design team's work. Despite the limitations discussed, the evidence review enabled colleagues with intellectual disabilities to make informed decisions about what to include in the final resource toolkit. The evidence review helped dispel the assumption that easy‐read end‐of‐care plans are by default accessible to people with intellectual disabilities and should therefore be a standard offer amongst services. It identified other creative approaches to EOLCP that the co‐design team believed to be more engaging and useful in terms of ‘prompting conversations’ (even if some of these resources proved very difficult to run through AGREE II). The evidence review also aided the co‐design group's decision to commission a number of new resources, which drew upon some of the most compelling features of identified material while integrating alternative content that the group felt was missing from the existing resources (notably the use of video and personal stories).

## 5 Conclusion and Recommendations

In this article, we have reflected on the methods and processes utilised for an evidence review that was used to inform the co‐design of an EOLCP toolkit for people with intellectual disabilities, while also considering its benefits and limitations. We anticipated in advance of commencing the study that relying on usual methods for identifying existing research, resources, tools and approaches was unlikely to reveal the breadth of material that was being used in everyday practice. In our experience, surveying those with experience supporting EOLCP for people with intellectual disabilities was the most fruitful method of identifying resources and tools currently in use, including in‐house tools that were unpublished and thus not publicly available. The survey was also valuable in highlighting the use of tools that were not specifically designed for people with intellectual disabilities but were being used by practitioners within intellectual disability services. On reflection, involving stakeholders from the intellectual disability community in promoting and circulating the survey (notably representatives from services and charitable organisations) was critical for achieving strong engagement.

The review of the academic literature, while useful for providing a comprehensive understanding of the topic, produced limited results in terms of identifying resources/approaches to EOLCP for people with intellectual disabilities. This was largely due to the fact that despite reference to end‐of‐life planning and decision‐making in the broader literature around end‐of‐life care for this population, detailed descriptions of the development and/or evaluation of approaches and resources for EOLCP for people with intellectual disabilities were largely absent.

Although AGREE II provided a useful starting point from which to appraise the material we identified, it proved to be quite limited for the purposes of supporting a review aimed specifically at supporting co‐design activities with an inclusive research team.

### 5.1 Recommendations

Our experience of conducting this evidence review has resulted in a number of recommendations. Some are specific to research and practice focused on EOLCP for people with intellectual disabilities; others have wider applicability to health and care research that is inclusive in its design:

- Further research is needed that evaluates existing approaches, tools and resources that are focused on EOLCP for people with intellectual disabilities.
- There is an urgent need for the co‐development of an appraisal tool specifically designed for the assessment of health and care resources aimed at people with intellectual disabilities and/or their carers. Alongside this, there is a need for a health and care appraisal tool that is designed specifically to be used by people with intellectual disabilities who are engaged in research and co‐design activities.
- We would welcome the development of a framework for conducting inclusive evidence reviews in health and care research that builds on the experimental practices outlined in this article.

### 5.2 Limitations

As discussed in this article, the quality appraisal process we adopted did not meet the needs of our research. For example, 16 resources/approaches mentioned in the academic literature were excluded due to insufficient information to run through AGREE. This may have skewed our perspective on currently available resources. It also indicates a possible inaccessibility of comprehensive information on EOLCP tools for people with intellectual disabilities.

## Author Contributions

**Elizabeth Tilley:** conceptualisation, investigation, funding acquisition, writing–original draft, methodology, writing–review and editing, formal analysis, validation. **Lorna Rouse:** investigation, writing–original draft, methodology, formal analysis. **Irene Tuffrey‐Wijne:** conceptualisation, investigation, funding acquisition, methodology, validation, writing–review and editing. **Rebecca Anderson‐Kittow:** conceptualisation, investigation, writing–review and editing, validation, funding acquisition.

## Ethics Statement

Ethics approval was given by the Open University Human Research Ethics Committee (application no. 4333) and Kingston University Research Ethics Committee (application no. 3015).

## Conflicts of Interest

The authors declare no conflicts of interest.

## Supporting information

Supporting information.

## Data Availability

Data sharing is not applicable to this article as no new data were created or analysed in this study.
