# Supplementary material for: Reflections on an Evidence Review Process to Inform the Co‐Design of a Toolkit for Supporting End‐of‐Life Care Planning With People With Intellectual Disabilities
Source: Health Expect. 2024 Oct 15;27(5):e70062. doi: 10.1111/hex.70062 (PMC11474664; doi:10.1111/hex.70062)
Supplement: Supplementary file 1 — Supporting information. [file HEX-27-e70062-s001.docx]

**Appendix S1**

Survey questions including responses for multiple choice questions

| **Question** | **Response** | | |
| --- | --- | --- | --- |
|  | **Yes** | **No** | **Not sure** |
| Have you (or your organisation) ever been involved with, and/or supported, someone with learning disabilities to **plan for their end-of-life care?**  *This may also be referred to as* ***advance care planning or future care planning.*** | 83 | 10 | 2 |
| Have you used any **tools, resources or guidelines** when supporting people with learning disabilities to do end-of-life care planning?  *These might be specific to end of life, or might be general decision-making tools that you use during end-of-life care planning.* | 64 | 12 | 9 |
| We are really interested to hear **how you/your organisation would go about** planning for the end of life with someone with learning disabilities. This doesn't have to be anything formal. Could you give an **example** of the way in which you have done this? Or tell us what approach you/your organisation would take, generally?  This might include:   - - **who** is involved in planning   - **when** planning takes place (i.e. only if a person is terminally ill, or earlier)   - **where** planning takes place   - **how** (i.e. what kinds of conversations you have).   You can write as much as you like (Please do NOT give any details that might identify individual people)  NOT EVERYONE USES GUIDELINES OR TOOLS, BUT IF YOU HAVE ANY LINKS TO RESOURCES YOU HAVE USED, PLEASE PROVIDE THEM HERE. * | 28 distinct resources (17 designed for working with people with learning disabilities, 11 generic) | | |
